# Effect of Pharmacist-Led Educational Intervention on Switching to Generic Medicine Among Patients Using Brand-Name Medicines in Japan

**DOI:** 10.3390/pharmacy14030070

**Published:** 2026-05-11

**Authors:** Tomohiko Tairabune, Terutaka Goto, Kentaro Yamazaki, Kenzo Kudo, Ken-Ichi Sako, Tomoji Maeda, Takeshi Chiba

**Affiliations:** 1 Regina Pharmacy Jimbocho, Unimat Life Co, Ltd., 1F Tamura Building 3-2-4 Kanda-Jimbocho, Chiyoda-ku, Tokyo 101-0051, Japan; 2 Division of Pharmacy, Unimat Life Co, Ltd., Unimat Aoyama Building 2-12-14 Minami-aoyama, Minato-ku, Tokyo 107-0062, Japan; 501786@unimatlife.jp; 3 Mayumi Pharmacy Shoto, Unimat Life Co, Ltd., 1F Matsuo Building 1-29-2 Shoto, Shibuya-ku, Tokyo 150-0046, Japan; 7816@unimatlife.jp; 4 Division of Clinical Pharmaceutics and Pharmacy Practice, Department of Clinical Pharmacy, School of Pharmacy, Iwate Medical University, 1-1-1 Idaidori, Yahaba-cyo 028-3694, Japan; kenzkudo@iwate-med.ac.jp; 5 Department of Clinical Pharmacology, Nihon Pharmaceutical University,10281 Komuro, Ina-Machi, Kitaadachi-gun, Saitama 362-0806, Japan; sakok@nichiyaku.ac.jp (K.-I.S.); t-maeda@nichiyaku.ac.jp (T.M.); 6 Department of Pharmacy, Juntendo University Hospital, 3-1-3 Hongo, Bunkyo-ku, Tokyo 113-8431, Japan; t.chiba.da@juntendo.ac.jp; 7 Laboratory of Clinical Pharmacology, Faculty of Pharmacy, Juntendo University, 6-8-1 Hinode, Urayasu-shi, Chiba 279-0013, Japan

**Keywords:** generic medicine, brand-name medicine, pharmacist, educational intervention, patient

## Abstract

The use of generic medicines (GEs) is being promoted to reduce healthcare costs. This study aims to evaluate the effect of a pharmacist-led educational intervention on the number of patients switching from brand-name medicines to GEs among patients who preferred brand-name products. Pharmacists provided a standardized explanation using pamphlets about GEs to patients who wished to use brand-name medicines and allocated time to answer questions. Basic knowledge about GEs (nine items) and perceptions of GE use (three items) were assessed before and after the intervention to determine whether changes influenced switching behavior. Between 1 March and 30 July 2025, 40 patients were enrolled in the analysis. Following the intervention, the number of patients using one or more brand-name medicines significantly decreased to 25. Knowledge and perceptions of GEs significantly increased after the intervention and were associated with an increased rate of switching to GEs. Binominal logistic regression analysis identified age ≥ 65 years as a strong factor associated with preference for brand-name medicines (OR, 16.45; 95% CI, 1.48–182.65; *p* = 0.02). These findings indicate that pharmacist-led educational interventions are effective in promoting GE use among patients who prefer brand-name medicines. In the future, we plan to conduct comparative studies with a control group to further investigate the factors that lead to patients strongly preferring the use of brand-name medicines.

## 1. Introduction

In Japan, the use of generic medicines (GEs) is being promoted to reduce healthcare costs associated with population aging and advances in medical care [1,2,3,4]. In 2013, the Ministry of Health, Labour and Welfare issued the “Roadmap for Further Promoting the Use of GE” [5] and has since implemented initiatives to encourage the use of GEs. As a result, GE utilization has increased annually; the volume share reached 85% in the September 2024 survey [6]. The Ministry has set a target of achieving a GE volume share of 80% or higher in all prefectures by the end of fiscal year 2029 and continues to promote GE use [7]. However, in value terms, GEs accounted for only 62.1% of total pharmaceutical expenditure in 2023, which remains relatively low compared with other countries [4,6]. Therefore, the government decided to achieve a new value term target of at least 65% by the end of fiscal year 2029 and reaffirmed its commitment to promoting the use of GEs [7].

A 2022 survey on the impact and implementation status of GE promotional measures reported that 48.8% of patients using brand-name medicines indicated “no specific reason” for not using GEs [8]. Previous studies have also shown that patients who prefer brand-name medicines often lack basic knowledge about GEs and have low positive perception regarding their use [9]. These findings suggest that patients do not have an adequate understanding of GEs.

Furthermore, a survey of patients using GEs found that the most common reason for switching from brand-name medicines was an “explanation from their family pharmacist” (36.2%). When combined with an “explanation from a pharmacist other than their family pharmacist”, pharmacist explanations accounted for 60.9% of responses [8]. These findings suggest patients may switch to GEs if pharmacists provide them information and educational intervention regarding GEs.

However, a prospective investigation of whether pharmacist-led educational interventions can effectively convince patients to switch from brand-name medicines to GEs remains lacking.

Therefore, this study aimed to investigate whether patients using brand-name medicines, who had limited knowledge and low perception of GEs, would switch from brand-name medicines to GEs following pharmacist-led educational intervention. Additionally, we compared the knowledge and perceptions of GEs before and after the intervention and assessed whether these findings contributed to an increase in the number of patients switching from brand-name medicines to GEs.

## 2. Materials and Methods

### 2.1. Study Design and Data Collection

**Figure 1:**
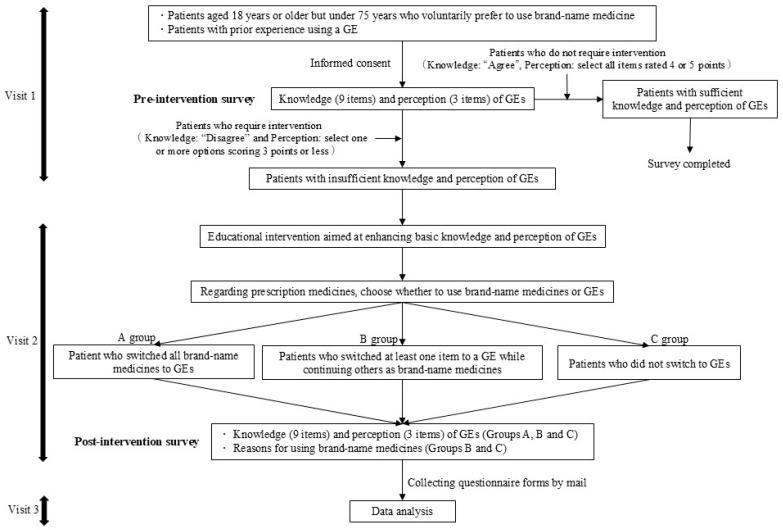
Survey process. GE: generic medicine.

Figure 1 illustrates the survey flow. Patients aged 18–75 years were enrolled between 1 March 2025 and 30 July 2025. Eligible patients were those who regularly visited our pharmacy, had prior experience using GEs, and were using brand-name medicines at their own request. Patients with mental disorders or cognitive impairments were excluded due to the potential risk of excessive distress. Participants were provided with written information regarding the study purpose, methods, preservation of anonymity, voluntary participation, and the absence of disadvantages for non-participation. Their responses were considered to provide consent to participate in the study.

**Figure 2:**
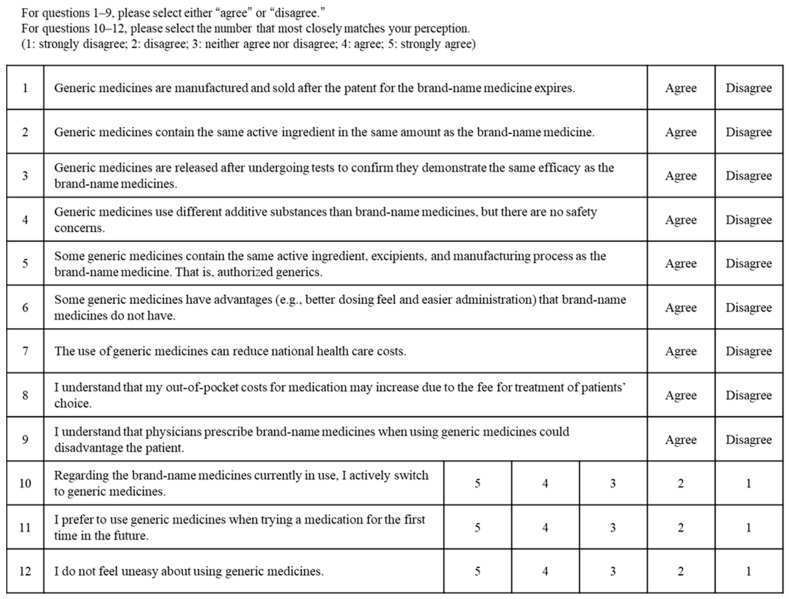
Questionnaire on knowledge and perceptions regarding GEs.

The questionnaire survey was conducted anonymously and administered before the pharmacist-led educational intervention at Visit 1 and after the intervention at Visit 2. The questionnaire comprised nine items evaluating basic knowledge of GEs and three items evaluating the perception of GE use (Figure 2). For the knowledge items, respondents selected either “agree” or “disagree”. Perception items were rated using a five-point Likert scale (1 = strongly disagree, 2 = disagree, 3 = neither agree nor disagree, 4 = agree, and 5 = strongly agree).

Furthermore, we investigated the reasons for continuing to use brand-name medicines after the educational intervention (multiple choice: having experienced reduced effectiveness when using a GE, having experienced side effects when using a GE, concerns about quality (drug content, expiration date, etc.), family or acquaintances not recommending GE use, concerns regarding GE distribution or supply, increased anxiety when switching to a GE due to changes in drug name or design, no particular reason, and other).

A questionnaire survey was conducted following the educational intervention on the same day. To reduce participant burden, the patients were asked to complete the questionnaire at home and return it in a self-addressed envelope.

### 2.2. Educational Interventions by Pharmacists

We determined eligibility for the educational intervention as follows. In the pre-intervention questionnaire survey, patients who answered “disagree” to questions regarding basic knowledge about GEs or who scored ≤3 on at least one question regarding perceptions of GE use were classified as having “insufficient knowledge and perceptions of GEs.” In this study, the educational intervention consisted of pharmacists providing standardized explanations using pamphlets about GEs to patients who preferred brand-name medicines and allocating time to answer questions. The content covered GEs’ manufacturing approval processes, efficacy, quality, safety, advantages not present in brand-name medicines [e.g., improved bitterness and dosing convenience], economic benefits of using GEs, the background of GE promotion policies, and fees related to patient preference for brand-name medicines. The standardized explanation and Q&A sessions were scheduled to take approximately 20 min.

However, the standardized explanation was delivered by multiple pharmacists, which posed a risk of variability in the intervention quality. To mitigate this risk, interventions were standardized using a checklist that comprehensively covered all essential items requiring explanation. Patients whose circumstances necessitated interruption during the explanation or a subsequent Q&A session were excluded from this study. The educational intervention was conducted in a separate consultation room within the pharmacy, rather than at the regular medication counseling counter, to protect the privacy of the participants.

### 2.3. Comparison of the Number of Patients Using Brand-Name Medicines, Knowledge, and Perception of GEs Before and After Pharmacist-Led Educational Intervention

The primary outcome was the number of patients using one or more brand-name medicines before and after the intervention. We compared the number of patients using one or more brand-name medicines before and after the intervention to determine whether switching from brand-name medicines to GEs increased. The secondary outcome assessed changes in basic knowledge of GEs and perceptions of their use, measured using a comprehensive evaluation index [10,11].

For the basic knowledge questions about GEs, responses of “agree” to each of the nine questions were considered correct, and the total number of correct responses (range, 0–9) was calculated for each patient. The mean number of correct responses (knowledge score) was then calculated for all patients and compared before and after the intervention. For the perception items, the mean response value for each patient was calculated based on the response scores (1–5) for the three questions. The overall mean response value (perception score) was subsequently calculated for all patients and compared before and after the intervention.

After the intervention, the patients were classified into three groups: Group A (switched all brand-name medicines to GEs), Group B (switched at least one item to a GE while continuing others as brand-name medicines), and Group C (did not switch to GEs). Within each group, pre- and post-intervention knowledge and perception scores were compared to evaluate differences in intervention effects among the groups.

### 2.4. Analysis of Factors Influencing the Preference for Brand-Name Medicines

Univariate analyses were conducted for age, sex, number of brand-name medicines used, prescription duration, number of currently used medications, and post-intervention knowledge and perception scores of GEs to identify factors associated with a strong preference for brand-name medicines after the intervention. Variables with *p* < 0.2 were selected as potential explanatory variables for multivariate analysis.

### 2.5. Statistical Analysis

Normality was assessed using normal probability plots and the Shapiro–Wilk test. Patient characteristics, including sex, age, therapeutic classes of brand-name medicines, number of brand-name medicines, prescription duration, and number of medicines used, were compared using the chi-square test or the Kruskal–Wallis test, as appropriate. The number of patients requesting brand-name medicines before and after the intervention was compared using the McNemar test. Knowledge and perception scores before and after the intervention were compared using the Wilcoxon signed-rank test. The factors associated with preference of patients for brand-name medicines were compared using a binomial logistic regression analysis. Statistical significance was considered at *p* < 0.05. All statistical analyses were performed using SPSS Statistics version 25 (IBM Corp., Armonk, NY, USA).

## 3. Results

### 3.1. Patient Characteristics

Table 1 lists the patient characteristics. A total of 47 patients were enrolled, and informed consent was obtained from all participants during the study period. Of these, 40 patients (22 males and 18 females) were included in the study after excluding 2 patients who did not submit the questionnaire and 5 patients for whom educational intervention could not be implemented according to the protocol. The valid response rate was 85%.

The average results were as follows: age, 56 years; prescription duration, 34 days; number of brand-name medicines used, 2.0; and number of medicines used, 2.8. Brand-name medicines were classified into 17 therapeutic categories, comprising 77 medicines. No significant differences in patient characteristics were observed for any of these items.

### 3.2. Comparison of the Number of Patients Using Brand-Name Medicines Before and After the Pharmacist-Led Educational Intervention

As shown in Table 2, the number of patients using brand-name medicines significantly decreased (*p* < 0.01) from 40 to 25 after the pharmacist-led educational intervention on GEs in patients using brand-name medicines.

### 3.3. Comparison of Knowledge and Perceptions of GEs Before and After the Pharmacist-Led Educational Intervention

**Figure 3:**
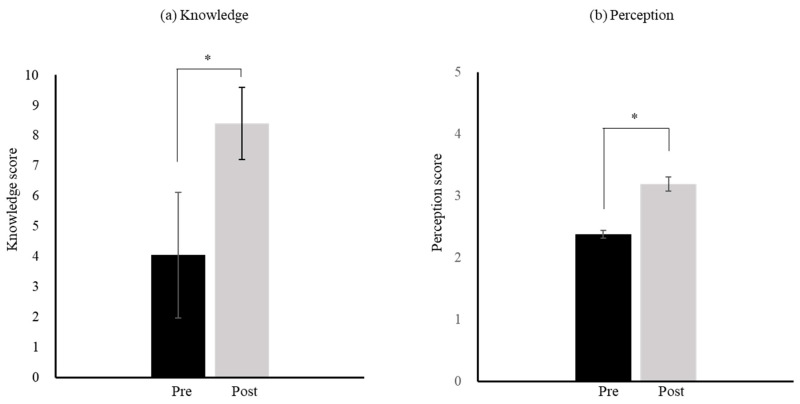
Comparison of knowledge and perception scores regarding GEs before and after pharmacist-led educational intervention. GE: generic medicine; Wilcoxon signed-rank test; * *p* < 0.05, *n* = 40. (**a**) The mean number of correct responses (knowledge score) was calculated for 40 participants and compared before and after the intervention. Mean ± SD. (**b**) The mean response value (perception score) was calculated for 40 participants and compared before and after the intervention. Mean ± SD.

Regarding the knowledge score, the mean number of correct responses per person was compared before and after the intervention. Knowledge scores were significantly higher after the intervention than before (8.4 vs. 4.1, *p* < 0.05) (Figure 3a).

Regarding the perception score, the mean response score per person was compared before and after the intervention. Perception scores were significantly higher after the intervention than before (3.2 vs. 2.4, *p* < 0.05) (Figure 3b). Moreover, the results of a comparison of the correct answer rates regarding knowledge and perception of GEs are provided in the Supplementary Materials (Supplementary Files S1 and S2).

### 3.4. Comparison of Knowledge and Perceptions of GEs Before and After Pharmacist-Led Educational Intervention: Classified by Whether GE Switching Occurred

**Figure 4:**
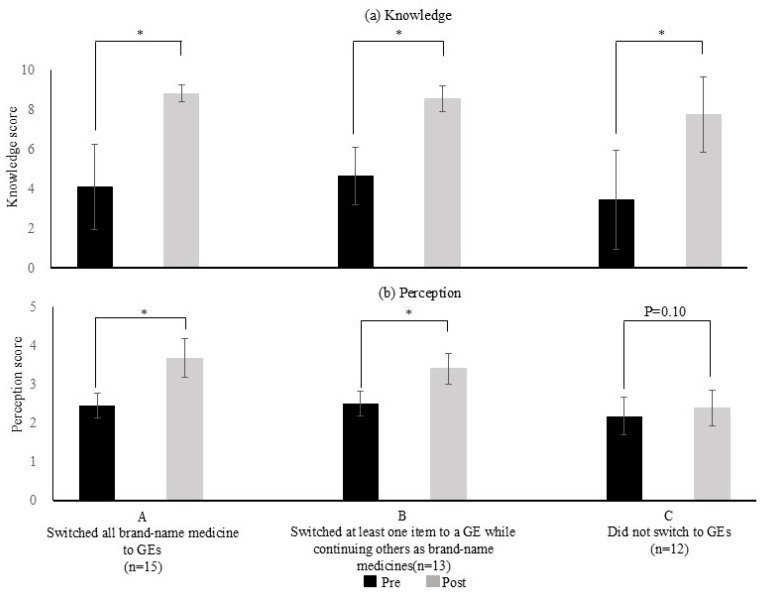
Comparison of knowledge and perception scores regarding GEs before and after pharmacist-led educational intervention: classified by whether GE switching occurred. GE: generic medicine; Wilcoxon signed-rank test; * *p* < 0.05, *n* = 40. (**a**) The average number of correct answers (knowledge score) was calculated for Groups A, B, and C and compared before and after the intervention. Mean ± SD. (**b**) The mean response values (perception scores) were calculated for Groups A, B, and C and compared before and after the intervention. Mean ± SD.

Following the intervention, the 40 patients were classified into three groups: Group A (15 patients), Group B (13 patients) and Group C (12 patients). As shown in Figure 4a, the post-intervention knowledge scores in Groups A, B, and C were 8.8 ± 0.1, 8.5 ± 0.2, and 7.8 ± 0.6, respectively, which were significantly higher than the corresponding pre-intervention scores of 4.1 ± 0.6, 4.6 ± 0.4, and 3.4 ± 0.7 (*p* < 0.05). In all groups, pharmacist intervention improved overall knowledge of GEs.

As shown in Figure 4b, the post-intervention perception scores in Groups A and B were 3.7 ± 0.1 and 3.4 ± 0.1, respectively, which were significantly higher than the pre-intervention scores of 2.4 ± 0.1 and 2.5 ± 0.1 (*p* < 0.05). In contrast, the perception scores in Group C showed no significant difference before and after the intervention (2.2 ± 0.1 vs. 2.4 ± 0.1, *p* = 0.10). Although the knowledge scores increased in Group C, the perception scores remained unchanged. Moreover, “the results of a comparison of correct answer rates with respect to knowledge and perception of GEs: Classified by whether GE switching occurred” are provided in the Supplementary Materials (Supplementary Files S3 and S4).

### 3.5. Multivariate Analysis of Factors Influencing the Preference for Brand-Name Medicines

Univariate analysis identified the following factors as potential explanatory variables for preference for brand-name medicines: age ≥ 65 years, use of ≥2 brand-name medicines, low overall knowledge of GEs after the intervention, and low perception of GEs after the intervention (Table 3a). Binomial logistic regression analysis identified age ≥ 65 years as a factor that is strongly associated with preference for brand-name medicines (OR, 16.45; 95% CI, 1.48–182.65; *p* = 0.02) (Table 3b).

### 3.6. Reasons for Using Brand-Name Medicines After Pharmacist Intervention

**Figure 5:**
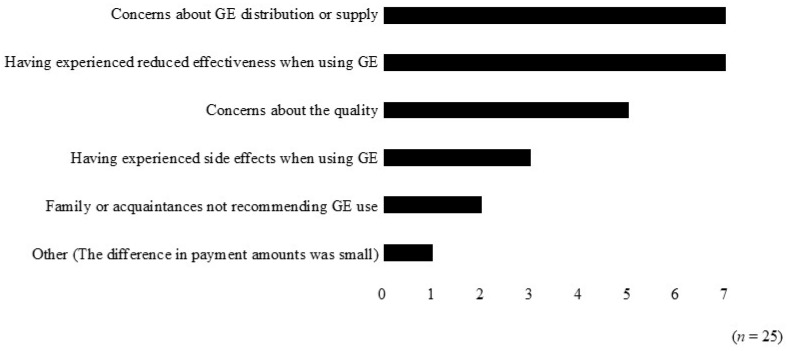
Reasons for using brand-name medicines after pharmacist-led educational intervention. GE: generic medicine.

After the intervention, a survey was conducted among the 25 patients who wished to continue using brand-name medicines to assess their reasons. The most common reasons were “Concerns about GE distribution or supply” and “Having experienced reduced effectiveness when using GEs,” each reported by 7 patients, followed by “Concerns about quality” (5 patients) and “having experienced side effects when using GE” (3 patients) (Figure 5).

## 4. Discussion

In Japan, the rules allow physicians to prescribe medications using both brand names and generic names. However, if a physician wishes to prescribe the brand-name medicines for any reason (such as a history of side effects or differences in indications between the brand-name and generic), they will include a note on the prescription stating that it cannot be substituted for a generic equivalent. Unless otherwise specified by the physician, the pharmacist will ask the patient whether they prefer the brand-name medicine or the GEs, confirm their preference, and then dispense the medication. In many cases, pharmacists advise patients to use GEs to help control medical costs.

We investigated whether pharmacist-led educational intervention on GEs would increase the number of patients switching from brand-name medicines to GEs. Furthermore, we investigated the patients’ knowledge and perceptions of GEs before and after the intervention and assessed whether these findings contributed to increasing the number of patients switching from brand-name medicines to GEs. In addition, we aimed to identify factors associated with continued use of brand-name medicines rather than GEs after the intervention. The number of patients using one or more brand-name medicines decreased from 40 to 25 following the intervention, suggesting its effectiveness (Table 2). To our knowledge, this study is the first to demonstrate that a pharmacist-led educational intervention targeting patients who request brand-name medicines increases switching to GEs. These findings indicate that pharmacists play a crucial role in encouraging patients who strongly prefer brand-name medicines to switch to a GE.

GE knowledge scores for all patients were significantly higher after than before the intervention. This finding suggests that the intervention improved the patients’ understanding of GEs and contributed to an increase in the number of patients switching from brand-name medicines to GEs (Figure 3a). In particular, the correct response rates for questions 4 (knowledge of additive substances in GEs) and 6 (knowledge of the advantages of GEs) were low before the intervention (15% for each). These results indicate that knowledge of these topics was insufficient among patients who preferred brand-name medicines and highlight the need for educational intervention.

Similarly, the GE perception scores were significantly higher after than before the intervention. This result suggests that the intervention improved the patients’ perceptions of GE use and contributed to increased switching from brand-name medicines to GEs (Figure 3b). A survey of Finnish pharmacists reported that patients’ insufficient knowledge of GEs hindered the medications’ promotion [12]. Previous studies have also suggested that educational and policy interventions by healthcare professionals targeting patients with insufficient knowledge or perceptions of GEs are essential to achieve the goal of promoting GE use [13,14,15,16,17]. Our study demonstrated that the intervention improved the patients’ basic knowledge of GEs, enhanced their perceptions of GE use and ultimately facilitated switching to GEs.

As shown in Figure 4a, the knowledge scores significantly increased in all groups after the intervention. This finding suggests that the pharmacist-led educational intervention improved patients’ overall knowledge of GEs, regardless of whether they subsequently switched to GEs. Furthermore, the perception scores in Groups A and B significantly increased after the intervention. In contrast, the patients in Group C did not show a significant increase in perception scores and did not switch to GEs (Figure 4b). In other words, although knowledge of GEs increased in Group C, perceptions regarding their use did not change, which may have prevented switching.

Multivariate analysis of the factors associated with preference for brand-name medicines identified age ≥ 65 years as a significant factor (Table 3). This finding suggests that patients aged ≥ 65 years who prefer brand-name medicines are likely to continue using them even after the intervention. Previous studies have reported that older adults who have used the same brand-name medicine for many years may refuse to switch to GEs due to a sense of safety associated with familiar medications [18,19,20]. Therefore, pharmacists should provide careful and reassuring explanations when discussing GE use with patients aged ≥ 65 years who prefer brand-name medicines to facilitate confident switching.

Furthermore, even after intervention, the most common reasons for continuing to use brand-name medicines were “Concerns about GE distribution or supply” and “Having experienced reduced effectiveness when using GEs” (Figure 5). In recent years, cases of GE shortages in Japan have increased due to (1) manufacturing suspensions resulting from penalties for deviations from approved manufacturing processes at GE companies and (2) reduced production volumes caused by difficulties in obtaining active pharmaceutical ingredients [21,22]. As a result, pharmacies have frequently been required to switch to alternative GEs or brand-name medicines for dispensing. Consequently, patients may have concerns regarding the distribution and supply of GEs. Strict adherence to the Generic Drug Supply Guidelines established in 2023 [23,24] may improve the supply of pharmaceuticals in Japan and enable a consistent supply of GEs, potentially alleviating patient concerns and improving perceptions of GE use.

Additionally, patients who have experienced reduced efficacy during previous GE use may develop negative perceptions regarding switching from brand-name medicines to GEs [18,25]. This study showed that many patients who wished to continue using brand-name medicines after the intervention expressed similar concerns.

This study has several limitations. First, it had a single-center design, and patients primarily from internal medicine departments were enrolled. Different results may be observed when patients from other departments are included. Second, this was a countywide comparative study without a control group, thus, the intervention group was not compared with a non-intervention control group. Consequently, factors such as differences in the timing of the intervention may have confounded the results. Finally, previous reports in Japan have indicated that patients with higher income tend to have lower GE usage rates [26,27]. However, this study did not assess patient income; therefore, this factor could not be examined.

## 5. Conclusions

When pharmacists provided educational intervention regarding GE use to patients requesting brand-name medicines, the number of patients who switched to GE increased. Furthermore, the intervention contributed to increased in the number of patients switching to GE by improving patients’ basic knowledge of GE and their perception of its use. Additionally, patients over 65 years of age may continue to prefer brand-name medicines even after intervention. To promote the use of generic medicines for medical cost containment, pharmacists should appropriately intervene in patients who prefer brand-name medicines and provide reassurance regarding GE use.

## Figures and Tables

**Table 1:** Baseline clinical characteristics of patients.

		All Patients (*n* = 40)		Switched All Brand-Name Medicines to GEs (*n* = 15)		Switched at Least One Item to a GE While Continuing Others as Brand-Name Medicines (*n* = 13)		Did not Switch to GEs (*n* = 12)		*p*-Value
Properties		*n*	%	*n*	%	*n*	%	*n*	%	
Sex	Male	22/40	55.0	9/15	60.0	5/13	38.5	8/12	66.7	0.33 ^a^
	Female	18/40	45.0	6/15	40.0	8/13	61.5	4/12	33.3	
Age	Mean ± SD	56.3 ± 13.9		50.7 ± 11.5		57.9 ± 14.7		61.4 ± 12.7		0.06 ^b^
	20s (20–29)	3/40	7.5	2/15	13.3	1/13	7.7	0/12	0.0	0.24 ^a^
	30s (30–39)	2/40	5.0	0/15	0.0	1/13	7.7	1/12	8.3	
	40s (40–49)	5/40	15.0	3/15	20.0	0/13	0.0	2/12	16.7	
	50s (50–59)	14/40	35.0	8/15	53.3	4/13	30.8	2/12	16.7	
	60s (60–69)	8/40	175	1/15	6.7	3/13	23.1	4/12	33.3	
	70 and older	8/40	20.0	1/15	6.7	4/13	30.8	3/12	25.0	
Therapeutic classes of brand-name medicines	Antihypertensive	18/77	23.4	6/27	22.2	5/27	18.5	7/23	30.4	0.11 ^a^
	Diabetes	5/77	6.5	2/27	7.4	0/27	0.0	3/23	13.0	
	Hyperlipidemia	10/77	13.0	3/27	11.1	6/27	22.2	1/23	4.3	
	Hyperuricemia	1/77	1.3	1/27	3.7	0/27	0.0	0/23	0.0	
	Hypnotic sedative	12/77	15.6	4/27	14.8	1/27	3.7	7/23	30.4	
	Antiarrhythmic	3/77	3.9	1/27	3.7	2/27	7.4	0/23	0.0	
	Proton pump inhibitor	5/77	6.5	2/27	7.4	2/27	7.4	1/23	4.3	
	Vertigo and balance disorders	1/77	1.3	0/27	0.0	1/27	3.7	0/23	0.0	
	Allergy	7/77	9.1	0/27	0.0	6/27	22.2	1/23	4.3	
	Corneal injury healing	1/77	1.3	1/27	3.7	0/27	0.0	0/23	0.0	
	Inhaled asthma and COPD	2/77	2.6	0/27	0.0	1/27	3.7	1/23	4.3	
	Urinary incontinence	1/77	1.3	0/27	0.0	1/27	3.7	0/23	0.0	
	Liver and gallbladder digestive	1/77	1.3	1/27	3.7	0/27	0.0	0/23	0.0	
	Treatment of mucosal defensive gastritis	1/77	1.3	1/27	3.7	0/27	0.0	0/23	0.0	
	Digestive function enhancer	2/77	2.6	2/27	7.4	0/27	0.0	0/23	0.0	
	Skin moisturization	1/77	1.3	0/27	0.0	1/27	3.7	0/23	0.0	
	NSAIDs	6/77	7.8	3/27	11.1	1/27	3.7	2/23	8.7	
Number of brand-name medicines Used	Mean ± SD	2.0 ± 1.0		1.6 ± 0.7		2.3 ± 0.8		1.9 ± 1.1		0.06 ^b^
	1	13/40	32.5	8/15	53.3	0/13	0.0	5/12	41.7	0.06 ^a^
	2	20/40	50.0	5/15	33.3	11/13	76.9	5/12	41.7	
	3	5/40	12.5	2/15	13.3	1/13	15.4	1/12	8.3	
	4≥	2/40	5.0	0/15	0.0	1/13	7.7	1/12	8.3	
Prescription Duration	Mean ± SD	34.3 ± 11.8		31.7 ± 9.9		37.0 ± 12.7		35.0 ± 13.1		0.54 ^b^
	Days 28–30	31/40	77.5	13/15	86.7	9/13	69.2	9/12	75.0	0.53 ^a^
	Days 56–60	9/40	22.5	2/15	13.3	4/13	30.8	3/12	25.0	
Number of medicines in use Mean ± SD		2.8 ± 1.6		3.0 ± 1.9		2.9 ± 1.2		2.8 ± 1.4		0.52 ^b^

^a^ χ^2^ test; ^b^ Kruskal–Wallis test; GE: generic medicine.

**Table 2:** Number of patients using brand-name medicines before and after pharmacist-led educational intervention.

		Post-Intervention			*p*-Value
		Using Brand-Name Medicines	Not Using Brand-Name Medicines	Total	
Pre-intervention	Using brand-name medicines	25	15	40	<0.01 *
	Not using brand-name medicines	0	0	0	
	Total	25	15	40	

McNemar’s test; * *p* < 0.05.

**Table 3:** (**a**) Univariate analysis of factors influencing the preference for brand-name medicines. (**b**) Multivariate analysis of factors influencing the preference for brand-name medicines.

(**a**)								
**Factor**			**Group That Switched Entirely to GEs After Intervention**	**Group Using One or More Brand-Name Medicines After Intervention**	**Odds Ratio**		**95% Confidence Interval**	***p*-Value**
			**(*n* = 15)**	**(*n* = 25)**				
Sex	Male		9	13	1			
	Female		6	12	1.63		0.44–5.95	0.46
Age	65<		14	12	1			
	65≥		1	13	15.17		1.72–133.53	0.01
Number of brand-name medicines used	2<		8	5	1			
	2≥		7	20	3.61		0.92–14.21	0.07
Prescription duration	30 days<		13	18	1			
	30 days≥		2	7	2.52		0.45–14.20	0.29
Number of medications in use	3<		8	12	1.00			
	3≥		7	13	1.23		0.34–4.46	0.74
Knowledge of GEs after intervention	High knowledge		12	14	1			
	Low knowledge		3	11	3.14		0.71–13.96	0.13
Perception of GEs after intervention	High perception		6	4	1			
	Low perception		9	21	3.50		0.79–15.48	0.10
(**b**)								
**Explanatory Variable**		**Odds Ratio**		**95% Confidence Interval**		***p*-Value**		
Age (65≥)		16.45		1.48–182.65		0.02 *		
Number of brand-name medicines used (2≥)		3.99		0.74–21.56		0.11		
Knowledge of GEs after intervention (low knowledge)		3.08		0.48–19.67		0.23		
Perception of GEs after intervention (low perception)		2.91		0.48–17.56		0.24		

GE: generic medicine; binomial logistic regression analysis; * *p* < 0.05, *n* = 40.

## Data Availability

The data presented in this study are available on request from the corresponding author due to privacy or ethical reasons.

## Supplementary Materials

The following supporting information can be downloaded at: https://www.mdpi.com/article/10.3390/pharmacy14030070/s1, File S1: Comparison of correct answer rates with respect to knowledge of GEs. File S2: Comparison of correct answer rates with respect to perception of GEs. File S3: Comparison of correct answer rates with respect to knowledge of GEs: Classified by whether GE switching occurred. File S4: Comparison of correct answer rates with respect to perception of GEs: Classified by whether GE switching occurred.

## Abbreviation

The following abbreviations are used in this manuscript:

GE	Generic medicine
