# Supplementary figures and images for: Effect of Pharmacist-Led Educational Intervention on Switching to Generic Medicine Among Patients Using Brand-Name Medicines in Japan
Source: Pharmacy (Basel). 2026 May 11;14(3):70. doi: 10.3390/pharmacy14030070 (PMC13214643; doi:10.3390/pharmacy14030070)

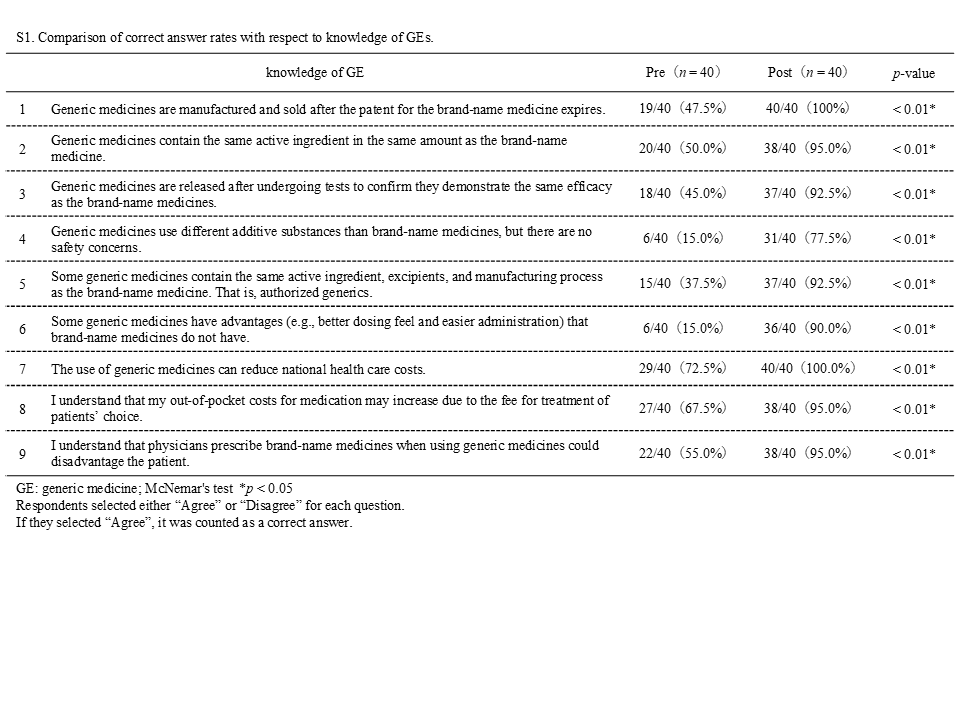

Supplement: Supplementary file 1 [file pharmacy-14-00070-s001.zip › Supplementary material/S1. Comparison of correct answer rates with respect to knowledge of GEs..TIF]

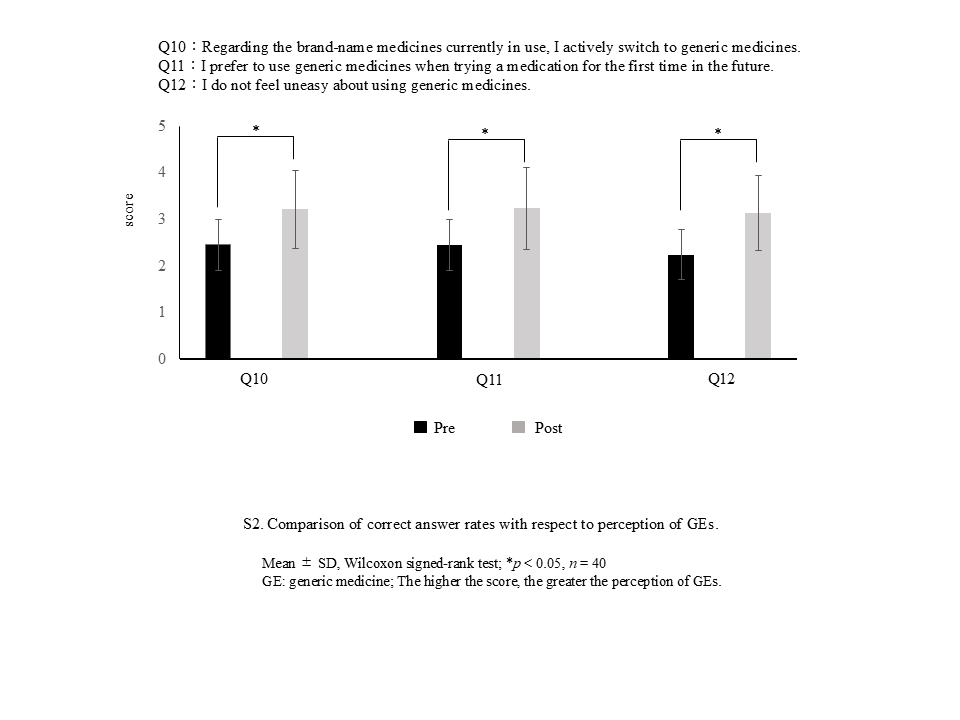

Supplement: Supplementary file 1 [file pharmacy-14-00070-s001.zip › Supplementary material/S2. Comparison of correct answer rates with respect to perception of GEs..TIF]

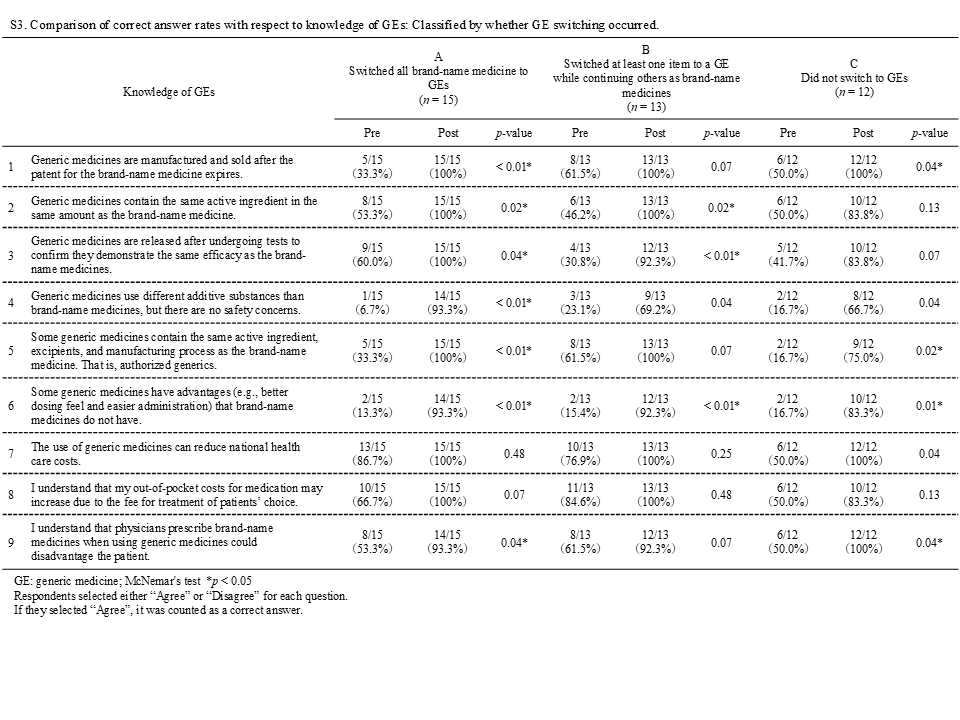

Supplement: Supplementary file 1 [file pharmacy-14-00070-s001.zip › Supplementary material/S3. Comparison of correct answer rates with respect to knowledge of GEs Classified by whether GE switching occurred..TIF]

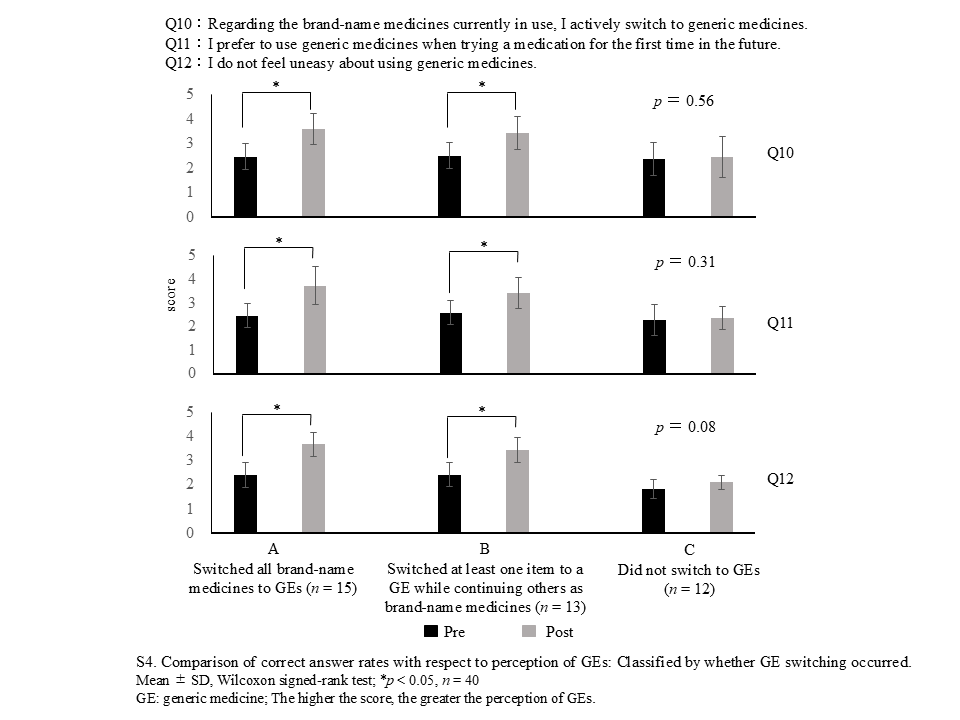

Supplement: Supplementary file 1 [file pharmacy-14-00070-s001.zip › Supplementary material/S4. Comparison of correct answer rates with respect to perception of GEs Classified by whether GE switching occurred..TIF]
